# Venous Thromboembolism Prophylaxis in Emergency General Surgery: A Retrospective Cohort Study of Timeliness, Prescribing, and Administration

**DOI:** 10.7759/cureus.115129

**Published:** 2026-08-24

**Authors:** Girishkumar Sivakumar, Manoj Nair

**Affiliations:** 1 General Surgery, North Middlesex University Hospital, Royal Free London NHS Foundation Trust, London, GBR; 2 UCL (University College London) Medical School, University College London, London, GBR; 3 Medical Education, Brighton and Sussex Medical School, Brighton, GBR; 4 Surgery, North Middlesex University Hospital, Royal Free London NHS Foundation Trust, London, GBR

**Keywords:** electronic prescribing, emergency general surgery, low-molecular-weight heparin, medication administration, patient safety, thromboprophylaxis, venous thromboembolism

## Abstract

Background

Venous thromboembolism (VTE) is a preventable cause of hospital-associated morbidity and mortality. In emergency general surgery, timely prophylaxis can be challenging because decisions regarding bleeding risk and urgent procedures must be balanced against the need for prompt pharmacological prophylaxis. This study quantified time to first pharmacological prophylaxis and compared VTE prevention processes across two sequential emergency general surgery cohorts.

Methods

We conducted a single-centre retrospective cohort study of adult emergency general surgical admissions across two predefined inpatient cohorts assessed approximately one month apart and separated by implementation of a targeted thromboprophylaxis practice change. Patient-level electronic prescribing and medication-administration records were used to measure time from admission to first low-molecular-weight heparin (LMWH) administration among patients for whom pharmacological prophylaxis was appropriate and a first administration time was assessable, and to evaluate VTE risk assessment, dose appropriateness, mechanical prophylaxis, and other process measures. Duplicate carry-over records, an elective admission, and records predating the implementation cutoff were excluded from the primary comparison. Categorical outcomes were compared using Fisher's exact test and continuous variables using the Mann-Whitney U test.

Results

Seventy-two emergency admissions were included (42 in the earlier cohort and 30 in the later cohort). Among patients with an assessable first LMWH administration time, the median admission-to-first-LMWH interval decreased from 20.8 hours (IQR: 13.9-27.9) to 16.4 hours (IQR: 10.5-21.5; p = 0.120), while administration within 14 hours increased from 8/32 (25.0%) to 9/24 (37.5%; p = 0.384). Correct completion of the electronic VTE risk-factor form increased from 8/42 (19.0%) to 26/30 (86.7%), an absolute increase of 67.6 percentage points (95% CI: 50.6-84.6; p < 0.001). Mechanical prophylaxis prescribing increased from 33/42 (78.6%) to 30/30 (100%; p = 0.008). Overall composite compliance did not change significantly.

Conclusions

In this retrospective cohort study, time to first LMWH administration improved numerically but remained frequently beyond the 14-hour threshold. Electronic VTE risk documentation and mechanical prophylaxis prescribing improved substantially in the later cohort. The findings identify a persistent gap between VTE assessment and timely pharmacological prophylaxis, highlighting medication-delivery processes as an important area for further evaluation and quality improvement in emergency general surgery.

## Introduction

Venous thromboembolism (VTE), encompassing deep-vein thrombosis and pulmonary embolism, is a major cause of preventable hospital-associated morbidity and mortality. Large international studies have demonstrated that a substantial proportion of hospitalised surgical patients are at risk of VTE and that recommended thromboprophylaxis remains incompletely implemented [1,2]. National guidance in the United Kingdom recommends that hospitalised patients are assessed for both VTE and bleeding risk and that pharmacological prophylaxis is started promptly when the risk of VTE outweighs the risk of bleeding [3,4]. Timeliness is clinically relevant: the National Institute for Health and Care Excellence (NICE) quality standard states that pharmacological prophylaxis should be started as soon as possible and within 14 hours of admission when indicated [4].

Emergency general surgery (EGS) presents particular challenges because acute inflammatory conditions, immobility, advanced age, trauma, emergency operations, active bleeding, neuraxial procedures, and uncertainty regarding operative timing may coexist within the same admission. A focused review of VTE prevention in EGS highlighted both the clinically important thrombotic risk in this population and the practical difficulty of applying prophylaxis guidance consistently across operative and non-operative pathways [5]. International guidance similarly supports pharmacological and/or mechanical prophylaxis for appropriately selected hospitalised surgical patients [6].

Previous studies have shown that structured quality-improvement strategies, electronic clinical decision support, audit and feedback, and targeted education can improve VTE risk assessment and prophylaxis prescribing [7-12]. However, prescription alone does not guarantee administration. Medication-administration studies have demonstrated that prescribed prophylaxis may be delayed or omitted, and missed enoxaparin doses have been associated with increased deep vein thrombosis in trauma and general surgery populations [13,14]. These findings support evaluating VTE prevention as a pathway extending beyond risk assessment and prescribing to actual medication administration.

Despite clear guidance, less is known about where delays occur between admission, VTE assessment, prescribing, and actual medication administration in emergency general surgery. We therefore undertook a patient-level retrospective cohort study across two sequential inpatient cohorts assessed approximately one month apart. The primary objective was to quantify the timeliness of first pharmacological thromboprophylaxis, principally the interval from admission to first low-molecular-weight heparin (LMWH) administration. Secondary objectives were to compare electronic risk assessment, prescribing, dose appropriateness, mechanical prophylaxis, and other VTE-prevention process measures between the two cohorts. We hypothesised that the later cohort would demonstrate shorter treatment delays and improved VTE-prevention processes.

## Materials and methods

Study design

This was a single-centre retrospective cohort study conducted within a high-volume emergency general surgery service in London, United Kingdom. Two sequential inpatient cohorts of routinely collected patient-level clinical, prescribing, and medication administration data were analysed approximately one month apart. Each cohort represented a predefined inpatient snapshot rather than a fixed admission window. The cohorts were separated by implementation of a targeted thromboprophylaxis practice change, allowing comparison of care processes before and after the intervention. The manuscript was prepared in accordance with relevant elements of the STROBE (Strengthening the Reporting of Observational Studies in Epidemiology) reporting framework for observational studies [15].

The source datasets contained 43 records in the earlier cohort and 45 records in the later cohort. One elective surgical admission was excluded because the study specifically evaluated the admission-to-assessment and admission-to-first-prophylaxis pathway in emergency general surgery. Elective surgical admissions follow a different planned perioperative pathway, including preoperative assessment, planned surgery, perioperative withholding of pharmacological prophylaxis, and postoperative reassessment, and were therefore not considered comparable for the primary timing outcomes. Eleven records in the later cohort represented patients whose ongoing admission had already been captured in the earlier cohort and were removed to avoid duplicate patient-level observations. Four additional records assigned to the later cohort had admission dates before the intervention implementation cutoff and were excluded from the primary later-cohort analysis to preserve temporal separation. The final comparative cohort therefore comprised 72 unique emergency admissions: 42 in the earlier cohort and 30 in the later cohort.

Participants

Eligible participants were adults under the emergency general surgery service who were inpatients at either of the two predefined cohort assessments and had electronic records sufficient to evaluate VTE risk assessment and relevant prophylaxis processes. Elective admissions were excluded. Duplicate patient records appearing across both cohorts were removed. For the later cohort, only admissions occurring after implementation of the practice intervention were included. Individual outcome denominators varied according to clinical eligibility for pharmacological prophylaxis. Patients were not included in the first LMWH timing analysis when LMWH was not administered because pharmacological prophylaxis was not appropriate at that stage of admission, for example, because of active gastrointestinal or rectal bleeding, trauma-related bleeding risk, imminent emergency surgery, or ongoing therapeutic anticoagulation where additional prophylactic LMWH was not required. No patient was excluded from the timing analysis because LMWH was clinically indicated but never administered; among patients for whom LMWH was indicated, the relevant outcome was the delay to the first administered dose.

Practice change between study cohorts

Between the two study cohorts, a targeted thromboprophylaxis practice change was implemented within the general surgery team. The intervention focused on improving complete electronic prescribing and medicines administration (EPMA) VTE risk-assessment documentation, appropriate LMWH selection and dosing, prompt prescription of mechanical prophylaxis when indicated, consideration of a stat first LMWH dose when clinically appropriate to avoid delay to the next routine medication round, and reassessment after surgery or significant clinical change. The intervention consisted of a concise guideline poster, targeted teaching and awareness within the clinical team, and direct feedback regarding recurring deficiencies identified in the earlier cohort. No structural modification was made to the EPMA system itself. The later cohort was assessed approximately one month after the earlier cohort, following implementation of these measures.

Variables and outcomes

Variables available across both study cohorts included age, sex, admission date and time, diagnosis, emergency/elective status, completion and timing of the EPMA VTE assessment, completeness of risk-factor documentation, whether pharmacological prophylaxis was indicated, LMWH prescribing, dose appropriateness, first LMWH administration time, contraindication documentation, mechanical prophylaxis prescribing, reassessment after surgery or clinical change, and an overall composite compliance assessment. Pharmacological prophylaxis was considered indicated when the documented VTE assessment supported LMWH use, and there was no contemporaneous clinical reason to withhold treatment. Accepted reasons for withholding pharmacological prophylaxis included active bleeding or clinically significant bleeding risk, immediate or imminent emergency surgery, trauma-related bleeding concerns, and other documented peri-operative or clinical contraindications. Patients receiving ongoing therapeutic anticoagulation were not considered to require additional prophylactic-dose LMWH unless their anticoagulation regimen had been modified as part of their inpatient management. Where admission, assessment, and administration timestamps were available, time intervals were recalculated directly from source timestamps rather than relying on manually entered delay fields. Mechanical prophylaxis was assessed according to whether it had been prescribed and, where recorded, whether prompt prescription was achieved. Although mechanical prophylaxis formed part of the overall VTE-prevention pathway and could contribute to composite non-compliance, the retrospective dataset did not contain a separate, consistently recorded patient-level variable adjudicating the indication for mechanical prophylaxis in every admission. This outcome was therefore reported as a prescribing process measure rather than as risk-adjusted appropriateness.

The primary outcome was time from admission to first LMWH administration among patients for whom pharmacological prophylaxis was clinically indicated and an administration timestamp was assessable. A prespecified clinically relevant secondary timing endpoint was first LMWH administration within 14 hours of admission, reflecting NICE guidance that indicated pharmacological prophylaxis should be started as soon as possible and within 14 hours of admission [4]. Additional secondary outcomes were first LMWH administration within 24 hours; time from completed VTE assessment to first LMWH administration; correct completion of the electronic VTE risk-factor assessment; mechanical prophylaxis prescribing; VTE assessment within 24 hours; correct LMWH dose among assessable prescriptions; and overall composite compliance where this could be determined. Overall composite compliance reflected correct completion of the VTE prophylaxis pathway, including appropriate completion of the electronic VTE assessment, prescription of pharmacological prophylaxis when indicated, correct LMWH dosing, timely administration of the first LMWH dose, and appropriate mechanical prophylaxis prescribing. Where a component was not clinically applicable, it was not treated as a compliance failure.

Data cleaning

The two source datasets were reconciled before analysis. Eleven records in the later cohort identified as patients already captured in the earlier cohort were removed. Dates and times were standardised, and assessment and LMWH delays were recalculated from admission and administration timestamps. Records assigned to the later cohort that predated the intervention implementation cutoff were excluded from the primary sequential-cohort comparison. No imputation was performed for missing or non-assessable outcomes; analyses used the available denominator for each endpoint.

Statistical analysis

Continuous variables were summarised using medians and interquartile ranges (IQRs) because the sample was modest and timing variables were not assumed to be normally distributed. Categorical variables were reported as counts and percentages. Categorical outcomes in the earlier and later cohorts were compared using Fisher's exact test. Continuous outcomes were compared using the two-sided Mann-Whitney U test. Mann-Whitney U statistics were reported for continuous comparisons. For selected binary outcomes, absolute risk differences with approximate 95% confidence intervals (CIs) were calculated. A two-sided p-value below 0.05 was considered statistically significant. The sample comprised all eligible unique emergency general surgical admissions identified in the two predefined inpatient cohort assessments. As this was a retrospective analysis of complete predefined cohorts rather than a prospectively recruited study, no a priori sample size calculation was performed, and the sample size was determined by the number of eligible patients available at the two assessments. Given the sample size and number of outcome events, the analysis was intentionally limited to descriptive statistics and unadjusted between-cohort comparisons rather than multivariable modelling.

Ethics and governance

The project was registered with the local Clinical Audit Department under reference NMUH059 (“VTE Prophylaxis in Acute General Surgical Admissions”). The study used routinely collected clinical, prescribing, and medication administration data, which were anonymised or pseudonymised for analysis. The NHS Health Research Authority decision tool confirmed that NHS Research Ethics Committee review was not required for sites in England (IRAS Project ID: 376247). No intervention outside routine clinical care was delivered to individual patients for the purposes of this study.

## Results

Cohort characteristics

The primary analysis included 72 emergency general surgical admissions: 42 in the earlier cohort and 30 in the later cohort. Median age was 62.5 years (IQR: 48.0-78.8) in the earlier cohort and 71.5 years (IQR: 44.5-81.8) in the later cohort (p = 0.549). Women comprised 20/42 (47.6%) and 18/30 (60.0%) of the cohorts, respectively (p = 0.345). These differences were not statistically significant. Baseline characteristics and outcome availability are summarised in Table 1.

**Table 1:** Baseline characteristics and outcome availability. IQR: interquartile range; VTE: venous thromboembolism; LMWH: low-molecular-weight heparin; U: Mann-Whitney U statistic; NA: not applicable. The Mann-Whitney U test was used for continuous comparisons, and Fisher's exact test was used for categorical comparisons.

Characteristic	Earlier cohort (n = 42)	Later cohort (n = 30)	Test statistic	p-value
Age, median (IQR), years	62.5 (48.0-78.8)	71.5 (44.5-81.8)	U = 577.0	0.549
Female sex, n (%)	20 (47.6%)	18 (60.0%)	Fisher's exact test	0.345
Assessable VTE assessment timing, n	41	30	NA	NA
Assessable first LMWH timing, n	32	24	NA	NA

VTE assessment and prophylaxis processes

Correct completion of the EPMA VTE risk-factor form improved markedly, from 8/42 (19.0%) in the earlier cohort to 26/30 (86.7%) in the later cohort. This represented an absolute increase of 67.6 percentage points (95% CI: 50.6-84.6; p < 0.001). Mechanical prophylaxis prescribing also improved from 33/42 (78.6%) to 30/30 (100%), an absolute increase of 21.4 percentage points (95% CI: 9.0-33.8; p = 0.008). Correct LMWH dose among assessable prescriptions increased from 29/32 (90.6%) to 24/24 (100%), although this did not reach statistical significance (p = 0.252).

VTE assessment itself was generally prompt in both cohorts. The median time from admission to assessment was 6.5 hours (IQR: 4.6-8.6) in the earlier cohort and 6.6 hours (IQR: 5.5-8.6) in the later cohort. All 41 patients in the earlier cohort with an assessable assessment time were assessed within 24 hours, compared with 29/30 (96.7%) in the later cohort (p = 0.423).

Timing of pharmacological prophylaxis

First LMWH administration timing was assessable in 32/42 admissions in the earlier cohort and 24/30 in the later cohort. Patients outside these denominators had not received LMWH because pharmacological prophylaxis was not appropriate at that stage of admission, including documented bleeding risk or imminent emergency surgery; there were no cases in which LMWH was clinically indicated but never administered. Among patients with an assessable first LMWH administration time, administration within 14 hours increased from 8/32 (25.0%) to 9/24 (37.5%), an absolute increase of 12.5 percentage points (95% CI: -12.0 to 37.0; p = 0.384). Administration within 24 hours increased from 20/32 (62.5%) to 18/24 (75.0%; p = 0.394). Median admission-to-first-LMWH time decreased from 20.8 hours (IQR: 13.9-27.9) to 16.4 hours (IQR: 10.5-21.5), but the difference was not statistically significant (p = 0.120). The median interval from completed VTE assessment to first LMWH administration decreased from 15.2 hours (IQR: 8.4-18.6) to 10.6 hours (IQR: 3.8-14.2; p = 0.070).

Overall composite compliance was assessable in 38 records in the earlier cohort and all 30 records in the later cohort. Compliance was 18/38 (47.4%) and 14/30 (46.7%), respectively (p = 1.000), indicating that substantial gains in selected process measures did not translate into improvement in the composite endpoint over the short interval between cohort assessments. The comparative VTE prevention outcomes are summarised in Table 2.

**Table 2:** Comparison of VTE prevention process measures between the earlier and later cohorts. EPMA: electronic prescribing and medicines administration; VTE: venous thromboembolism; LMWH: low-molecular-weight heparin; IQR: interquartile range; CI: confidence interval; pp: percentage points; U: Mann-Whitney U statistic. Fisher's exact test was used for categorical comparisons, and the Mann-Whitney U test for continuous comparisons.

Outcome	Earlier cohort	Later cohort	Absolute difference	Test statistic	p-value
Correct EPMA VTE risk-factor form, n/N (%)	8/42 (19.0%)	26/30 (86.7%)	+67.6 pp (95% CI: 50.6 to 84.6)	Fisher's exact test	<0.001
Mechanical prophylaxis prescribed, n/N (%)	33/42 (78.6%)	30/30 (100%)	+21.4 pp (95% CI: 9.0 to 33.8)	Fisher's exact test	0.008
Correct LMWH dose, n/N (%)	29/32 (90.6%)	24/24 (100%)	+9.4 pp	Fisher's exact test	0.252
VTE assessment within 24 hours, n/N (%)	41/41 (100%)	29/30 (96.7%)	-3.3 pp	Fisher's exact test	0.423
Admission to VTE assessment, median (IQR), hours	6.5 (4.6-8.6)	6.6 (5.5-8.6)	+0.1 hours	U = 619.0	0.968
First LMWH within 14 hours, n/N (%)	8/32 (25.0%)	9/24 (37.5%)	+12.5 pp (95% CI: -12.0 to 37.0)	Fisher's exact test	0.384
First LMWH within 24 hours, n/N (%)	20/32 (62.5%)	18/24 (75.0%)	+12.5 pp	Fisher's exact test	0.394
Admission to first LMWH, median (IQR), hours	20.8 (13.9-27.9)	16.4 (10.5-21.5)	-4.4 hours	U = 478.5	0.120
VTE assessment to first LMWH, median (IQR), hours	15.2 (8.4-18.6)	10.6 (3.8-14.2)	-4.5 hours	U = 494.0	0.070
Overall composite compliance, n/N (%)	18/38 (47.4%)	14/30 (46.7%)	-0.7 pp	Fisher's exact test	1.000

## Discussion

This retrospective cohort study of two sequential emergency general surgery inpatient cohorts identified a persistent delay between admission and delivery of pharmacological VTE prophylaxis. Median time to first LMWH administration improved from 20.8 to 16.4 hours, and the proportion receiving LMWH within 14 hours increased from 25.0% to 37.5%, although neither difference reached statistical significance in this modest sample. In parallel, electronic VTE risk-factor documentation and mechanical prophylaxis prescribing improved substantially. The divergence between these findings is clinically informative: processes centred on assessment and prescribing appeared more responsive to the practice change than the downstream medication delivery interval.

The most pronounced improvement was in correct completion of the EPMA VTE risk-factor form, which increased from 19.0% to 86.7%. Previous work has demonstrated that active implementation strategies, including clinical decision support, audit and feedback, individualised performance feedback, and multidisciplinary VTE prevention programmes, can improve risk-appropriate prophylaxis prescribing and adherence to recommended practice [7-12,16-22]. In particular, individualised feedback to surgical trainees has been associated with improved appropriate prophylaxis prescribing, while broader institutional programmes have demonstrated the value of combining education, electronic systems, measurement, and feedback rather than relying on passive guideline dissemination alone [17-20]. Our findings are consistent with this literature and suggest that the quality and completeness of an electronic risk assessment, rather than simply whether an assessment has been recorded, are important process measures.

Mechanical prophylaxis prescribing increased from 78.6% to 100% in the later cohort. This improvement is consistent with the broader principle that structured protocols and active implementation strategies can improve adherence to VTE prevention processes [7,8,17]. Nevertheless, mechanical prophylaxis should not be considered interchangeable with pharmacological prophylaxis. Selection of prophylactic measures should remain individualised according to VTE risk, bleeding risk, mobility, operative status, and contraindications, in accordance with national and international guidance [3,6]. Accordingly, the observed improvement reflects prescribing practice rather than a formal patient-level assessment of the appropriateness of mechanical prophylaxis in every case.

The pharmacological prophylaxis findings were more nuanced. Only one quarter of patients with an assessable administration time received their first LMWH dose within 14 hours in the earlier cohort, increasing to 37.5% in the later cohort. Median admission-to-first-LMWH time decreased by approximately 4.4 hours, while the median interval from completed VTE assessment to first LMWH administration decreased by approximately 4.5 hours. Neither difference reached statistical significance, although the latter approached significance. These findings indicate that a substantial component of the observed interval to first LMWH occurred after completion of the VTE assessment. The specific reasons for this delay were not directly recorded in the study; possible contributors include routine medication administration schedules, peri-operative decision-making, bleeding concerns, and the absence of an immediate first-dose prescription.

The distinction between prescribing prophylaxis and actually administering it is increasingly recognised as an important patient-safety issue. Studies using medication administration data have demonstrated substantial non-administration of prescribed pharmacological VTE prophylaxis [13,23]. Missed enoxaparin doses have been associated with increased deep vein thrombosis in trauma and general surgery patients [14]. In patients with traumatic spinal cord injury, missed prophylaxis doses have also been investigated as a potentially modifiable contributor to thrombotic risk [24]. More recent trauma data have reinforced the importance of timely initiation, with longer admission-to-prophylaxis intervals associated with subsequent VTE, supporting the concept of time to prophylaxis as a measurable quality metric [25]. Although these populations are not identical to emergency general surgery, they provide supporting evidence that assessment and prescription alone do not fully characterise the quality of thromboprophylaxis delivery.

Our findings suggest that clinician-facing education and improved documentation may be insufficient on their own to address all delays in pharmacological prophylaxis administration. Previous studies have shown that electronic decision support, patient education, and audit-and-feedback strategies can improve VTE prevention processes when integrated into clinical workflow [9-12,16,18-22,26]. In emergency general surgery, further quality-improvement work could therefore evaluate medication timing defaults, appropriate stat first-dose prescribing, clearer peri-operative hold-and-restart instructions, and closer medical-nursing review of delayed or omitted doses.

The absence of improvement in the overall composite compliance measure despite substantial improvement in selected domains should not be interpreted as evidence that no meaningful process change occurred. Composite measures incorporate several independent opportunities for non-compliance, meaning that improvement in risk documentation or mechanical prophylaxis may not alter the overall classification if another component remains unmet. In this study, persistent delays to first LMWH administration and non-compliance in other recorded components of the VTE prophylaxis pathway limited improvement in the composite endpoint. Reporting the individual components alongside the composite measure therefore provides a more informative description of where practice improved and where further intervention is required.

This study has several strengths. Patient-level electronic timestamps were used to quantify the pathway from admission through VTE assessment to first LMWH administration, allowing timeliness to be evaluated as a measurable clinical process rather than solely as a binary prescribing or documentation endpoint. Duplicate patient observations were removed, the later cohort was restricted to admissions occurring after the implementation cutoff, and both statistically significant and non-significant findings are reported. The sequential cohort design also permits assessment of whether changes in practice were accompanied by measurable changes in care delivery.

There are important limitations. This was a single-centre retrospective observational study with a modest sample size and a short interval between cohorts. The analysis was restricted to variables available in routinely collected clinical, prescribing, and medication administration records, limiting adjustment for potential case-mix differences and precluding reliable multivariable modelling. Individual outcome denominators varied because pharmacological prophylaxis could appropriately be withheld because of bleeding risk, therapeutic anticoagulation, urgent surgery, or other clinical considerations, and some records lacked an assessable administration timestamp. The study was not powered for patient-level clinical outcomes such as hospital-acquired VTE, pulmonary embolism, major bleeding, or mortality; the findings should therefore be interpreted as process outcomes rather than evidence of reduced clinical-event rates. The sequential pre/post design is also susceptible to temporal confounding and the Hawthorne effect, while the multifaceted nature of the practice change prevents attribution of observed improvements to any single component.

Future work should focus particularly on the persistent admission-to-administration delay. Potential interventions include standardised stat-first-dose prescribing when clinically appropriate, electronic prompts linked to admission and medication timing, clearer peri-operative hold-and-restart instructions, automatic reassessment prompts after surgery or clinical change, and joint medical-nursing feedback concerning missed or delayed doses. Larger prospective studies conducted over longer sampling periods and, ideally, across multiple high-volume emergency general surgery centres would allow recruitment of larger cohorts, better adjustment for case-mix differences, and evaluation of patient-level outcomes such as hospital-acquired VTE, major bleeding, and mortality. Such studies could also determine whether targeted workflow interventions improve timely pharmacological prophylaxis without increasing bleeding complications.

## Conclusions

In this retrospective cohort study of emergency general surgical admissions, time to first LMWH administration improved numerically between sequential study cohorts but remained frequently beyond the 14-hour treatment threshold. Correct electronic VTE risk factor documentation and mechanical prophylaxis prescribing improved substantially, while the change in pharmacological prophylaxis timing was smaller and not statistically significant.

These findings suggest a possible gap between assessment and timely prophylaxis delivery. Further quality-improvement work should evaluate medication timing processes alongside accurate risk assessment and prescribing, including strategies that reduce avoidable waits for the next routine administration round while preserving appropriate clinical exceptions for bleeding risk, urgent surgery, and therapeutic anticoagulation.
